# Aortic pulse wave velocity assessment in CMR: a novel method for transit time estimation

**DOI:** 10.1186/1532-429X-15-S1-E25

**Published:** 2013-01-30

**Authors:** Antonella Meloni, Heather M Zymeski, Alessia Pepe, Massimo Lombardi, John C Wood

**Affiliations:** 1 CMR Unit, Fondazione G.Monasterio CNR-Regione Toscana and Institute of Clinical Physiology, Pisa, Italy; 2 Department of Pediatrics, Division of Cardiology, Children's Hospital Los Angeles, Los Angeles, CA, USA; 3 Department of Radiology, Children's Hospital Los Angeles, Los Angeles, CA, USA

## Background

Aortic pulse wave velocity (PWV) is considered as the "gold standard" measurement of arterial stiffness and is commonly calculated as the ratio between the distance separating two locations along the artery and the transit time (Δt) needed for the pressure or velocity wave to cover this distance. PWV is increasingly assessed by means of cardiovascular magnetic resonance (CMR). Our goal was evaluate the efficiency of a novel method for Δt estimation, based on the principle of group delay (TT-GD method).

## Methods

Flow curves were estimated from phase contrast (PC) images of 30 patients. The TT-GD method operates in the frequency domain and models the ascending aortic waveform as an input passing through a discrete-component "filter", producing the observed descending aortic waveform, so that the group delay (GD) of that filter represents the average time-delay. This method was compared with two previously described time-domain methods: TT-point using the half-maximum of the curves and TT-wave using cross correlation. In order to study the effect of the temporal resolution on ΔT estimates, the original flow curves were downsampled of a factor of two, three and four.

## Results

Mean Δts obtained with the three methods were comparable (TT-GD: 28.18±5.36 ms, TT-point: 27.02±5.32 ms, TT-wave: 26.93±4.41; P=0.561).

The TT-GD method was the most robust to reduced temporal resolution (Table 1).

**Table 1:** Influence of temporal resolution on the Δts estimated from flow curves.

	Downsampling by 2	Downsampling by 3	Downsampling by 4
**TT-GD**			

***Best-fitting line: slope Intercept (ms)***	1.007 ± 0.047 -0.271 ± 1.361	0.929 ± 0.063 1.891 ± 1.810	0.923 ± 0.088 3.131 ± 2.519

***R-squared for the linear fitting***	0.941	0.886	0.798

***Difference, mean ± SD (ms)***	-0.08 ± 1.34	-0.10 ± 1.83	0.97 ± 2.52

***P (paired test)***	0.746	0.756	0.074

***CoV (%)***	3.33	4.52	6.57

***ICC***	0.985	0.970	0.937

***Correlation, r (P-value)***	0.970 (P<0.0001)	0.941 (P<0.0001)	0.893 (P<0.0001)

***BA limits (ms)***	-2.7 to 2.6	-3.7 to 3.5	-4.0 to 5.9

**TT-POINT**			

***Best-fitting line: slope Intercept (ms)***	0.722 ± 0.156 7.483 ± 4.287	0.927 ± 0.187 1.469 ± 5.147	0.506 ± 0.215 15.093 ± 5.921

***R-squared for the linear fitting***	0.434	0.467	0.165

***Difference, mean ± SD (ms)***	-0.03 ± 4.63	-0.52 ± 5.28	1.74 ± 6.6

***P (paired test)***	0.971	0.597	0.159

***CoV (%)***	11.91	13.78	17.04

***ICC***	0.798	0.794	0.559

***Correlation, r (P-value)***	0.659 (P<0.0001)	0.683 (P<0.0001)	0.406 (P=0.026)

***BA limits (ms)***	-9.1 to 9.0	-10.9 to 9.8	-11.2 to 14.7

**TT-WAVE**			

***Best-fitting line: slope Intercept (ms)***	0.917 ± 0.084 1.637 ± 2.303	0.926 ± 0.096 1.871 ± 2.622	0.809 ± 0.108 6.175 ± 2.939

***R-squared for the linear fitting***	0.808	0.768	0.668

***Difference, mean ± SD (ms)***	-0.61 ± 2.01	-0.13 ± 2.27	1.04 ± 2.66

***P (paired test)***	0.108	0.757	0.040

***CoV (%)***	5.49	5.89	7.24

***ICC***	0.944	0.935	0.889

***Correlation, r (P-value)***	0.899 (P<0.0001)	0.876 (P<0.0001)	0.818 (P<0.0001)

***BA limits (ms)***	-4.6 to 3.3	-4.6 to 4.3	-4.2 to 6.2

While the TT-GD as well as the TT-wave produced comparable results for velocity and flow waveforms (coefficient of variability or CoV: 4.81% and 5.04, respectively), the TT-point resulted in significant shorter Δt values when calculated from velocity waveforms (CoV=8.71%, mean difference: 1.78±2.73 ms).

The TT-GD method was the most reproducible, with an intra-observer variability of 3.38% and an inter-observer variability of 3.67%.

## Conclusions

Since the TT-GD method operates in the frequency domain, it was more robust to reduced temporal resolution than either of the time-domain methods. Moreover, it was more robust to the waveform type and more reproducible.

## Funding

This work was part of National Institutes of Health trial supported by the National Heart Lung and Blood Institute grant # 1 RO1 HL075592-01A1.

